# Combined effects of genotype and childhood adversity shape variability of DNA methylation across age

**DOI:** 10.1038/s41398-020-01147-z

**Published:** 2021-02-01

**Authors:** Darina Czamara, Elleke Tissink, Johanna Tuhkanen, Jade Martins, Yvonne Awaloff, Amanda J. Drake, Batbayar Khulan, Aarno Palotie, Sibylle M. Winter, Charles B. Nemeroff, W. Edward Craighead, Boadie W. Dunlop, Helen S. Mayberg, Becky Kinkead, Sanjay J. Mathew, Dan V. Iosifescu, Thomas C. Neylan, Christine M. Heim, Jari Lahti, Johan G. Eriksson, Katri Räikkönen, Kerry J. Ressler, Nadine Provençal, Elisabeth B. Binder

**Affiliations:** 1 Department of Translational Research in Psychiatry, Max Planck Institute of Psychiatry, Kraepelinstr. 2-10, 80804 Munich, Germany; 2 Department of Complex Trait Genetics, Center for Neurogenomics and Cognitive Research, Amsterdam Neuroscience, Vrije Universiteit Amsterdam, 1081 HV Amsterdam, The Netherlands; 3 Department of Psychology and Logopedics, Faculty of Medicine, University of Helsinki, Haartmaninkatu 3, 00014 Helsinki, Finland; 4 Sopra Steria, 80804 Munich, Germany; 5 University/British Heart Foundation Centre for Cardiovascular Science, Queen’s Medical Research Institute, Edinburgh BioQuarter, University of Edinburgh, 47 Little France Crescent, Edinburgh, EH16 4TJ UK; 6 Institute for Molecular Medicine Finland (FIMM), University of Helsinki, 00014 Helsinki, Finland; 7 Department of Child and Adolescent Psychiatry, Charité—Universitätsmedizin Berlin, Campus Virchow, 13353 Berlin, Germany; 8 Department of Psychiatry, Dell Medical School, University of Texas at Austin, 1601 Trinity St, Austin, TX 78712 USA; 9 Department of Psychiatry and Behavioral Sciences, Emory University School of Medicine, 12 Executive Park Dr, Atlanta, GA 30329 USA; 10 Icahn School of Medicine at Mount Sinai, 1 Gustave L. Levy PI, New York, NY 10029 USA; 11 Menninger Department of Psychiatry and Behavioral Sciences, Baylor College of Medicine Mental Health Care Line, Michael E. Debakey VA Medical Center, Houston, TX USA; 12 NYU School of Medicine and Nathan Kline Institute, New York, NY USA; 13 Departments of Psychiatry and Neurology, University of California, San Francisco, CA USA; 14 Charité–Universitätsmedizin Berlin, Corporate Member of Freie Universität Berlin, Humboldt-Universität zu Berlin, Berlin Institute of Health (BIH), Institute of Medical Psychology, Luisenstraße 57, 10117 Berlin, Germany; 15 Turku Institute for Advanced Studies, University of Turku, 20500 Turku, Finland; 16 Department of General Practice and Primary Health Care, Helsinki University Hospital, University of Helsinki, 00290 Helsinki, Finland; 17 Folkhälsan Research Center, 00250 Helsinki, Finland; 18 Department of Obstetrics and Gynecology, Yong Loo Lin School of Medicine, National University of Singapore, Singapore, Singapore; 19 Singapore Institute for Clinical Sciences, Singapore, Singapore; 20 Mailman Research Center, 115 Mill St., Mailstop 339, Belmont, MA 02478 USA; 21 Faculty of Health Sciences, Simon Fraser University, 8888 University Drive, Burnaby, BC Canada; 22 BC Children’s Hospital Research Institute, Vancouver, BC Canada

**Keywords:** Psychiatric disorders, Psychology

## Abstract

Lasting effects of adversity, such as exposure to childhood adversity (CA) on disease risk, may be embedded via epigenetic mechanisms but findings from human studies investigating the main effects of such exposure on epigenetic measures, including DNA methylation (DNAm), are inconsistent. Studies in perinatal tissues indicate that variability of DNAm at birth is best explained by the joint effects of genotype and prenatal environment. Here, we extend these analyses to postnatal stressors. We investigated the contribution of CA, *cis* genotype (G), and their additive (G + CA) and interactive (G × CA) effects to DNAm variability in blood or saliva from five independent cohorts with a total sample size of 1074 ranging in age from childhood to late adulthood. Of these, 541 were exposed to CA, which was assessed retrospectively using self-reports or verified through social services and registries. For the majority of sites (over 50%) in the adult cohorts, variability in DNAm was best explained by G + CA or G × CA but almost never by CA alone. Across ages and tissues, 1672 DNAm sites showed consistency of the best model in all five cohorts, with G × CA interactions explaining most variance. The consistent G × CA sites mapped to genes enriched in brain-specific transcripts and Gene Ontology terms related to development and synaptic function. Interaction of CA with genotypes showed the strongest contribution to DNAm variability, with stable effects across cohorts in functionally relevant genes. This underscores the importance of including genotype in studies investigating the impact of environmental factors on epigenetic marks.

## Introduction

Childhood adversity (CA), including child abuse and neglect, is a major risk factors for the development of stress-related psychiatric and other medical disorders later in life^1–4^. Exposure to CA is not only associated with disease risk, but also with a number of lasting biological and physiological changes, including alterations in brain structure, function, and connectivity^5^, stress response^6^, and immune function^7^.

DNA methylation (DNAm) has been proposed as a biological process by which early-life adversity may have lasting effects on gene transcription providing a molecular mechanism for how early environment could influence health outcomes later in life^8,9^. A number of studies have investigated DNAm changes with exposure to CA in peripheral tissues, such as saliva or blood, either using candidate gene approaches or genome-wide DNAm studies (EWAS). Overall, while there is some evidence for the association between CA and altered patterns of DNAm, results for individual DNAm targets remain inconsistent^10^.

The majority of autosomal CpGs (about 80%) are not variable across tissues and individuals^11,12^, leaving only about 20% of CpG sites that may contribute to differences in phenotypes and health^13^. These variable CpGs are of specific interest as they are enriched for functionally relevant genomic regions, associated with effects on gene expression^12^. In contrast to CA, genetic factors have been shown to have replicable influences on DNAm variability. The impact of genetic variation, especially of single-nucleotide polymorphisms (SNPs), on DNAm in different tissues, has been investigated in many studies and a large number of methylation quantitative trait loci (SNPs significantly associated with DNAm status^14^) have been discovered which are relatively stable throughout the life course^15^.

Environmental factors and genetic factors may thus act in concert to influence DNAm, however only a few studies have investigated the joint effects of environment and genotype on DNAm variability. In the context of the influence of prenatal environments on DNAm at birth, Teh et al.^16^ as well as our group^17^ reported that combined effects of genotype and prenatal environment explain most of the variance in umbilical cord and cord blood DNAm. In fact, environment alone was almost never the strongest driver, rather additive or interactive effects of genotype (G) and environment (E) explained DNAm variability best in the majority of CpGs. This may be specific of prenatal environments, where there is less time for exposure.

Here, we aimed to expand the analysis of combined G and E effects to a postnatal stressor (CA). We examined if, similar to our results in neonates, combined effects are also stronger drivers of DNAm variation later in life and if the proportion of explained variance varies with time to exposure, i.e., whether effects of CA measured in childhood are qualitatively or quantitatively different than when measured later in life. For this purpose, we systematically tested main effects of CA (E = CA) and genotype located in a 1 MB window of the CpG (G) on DNAm as well as their additive (G + CA) and multiplicative effects (G × CA). For each tested CpG site, we sought the model that explained most of the DNAm variability. We explored this in five independent cohorts with a total of 1074 individuals, of whom 541 were exposed to CA. The five cohorts ranged in age from early childhood (3–5 years of age) to elderly individuals (mean age of 64 years) with both retrospective self-reports of CA and verified exposures by registries or social services. This enabled us to test for the stability of G and CA effects with age as well as across different types of assessment of exposure.

## Methods

### Samples

Five independent cohorts were included in our analysis: GRADY, PReDICT, U19, BerlinLCS, and HBCS. All subjects (or their legal guardians) gave written informed consent and ethical approval was given by the Institutional Review Board or Ethical Committee of each site participating in every study. Register linkage has been conducted with permission from the register authority (HBCS: the Finnish National Archives).

The GRADY cohort consisted of 309 participants who were recruited as part of the GRADY Trauma Project at the Grady Memorial Hospital in Atlanta, Georgia^18^. All participants come from an urban population with low socioeconomic status and are characterized by high prevalence and severity of trauma over lifetime^19–21^.

The PReDICT cohort consisted of 363 treatment-naive patients who met criteria for current major depressive disorder. All participants were recruited at three Atlanta sites associated with the Emory University School of Medicine, Department of Psychiatry and Behavioral Sciences^22^.

The U19 cohort consisted of 78 nonmedicated women recruited at four academic sites in the USA (Emory University, Icahn School of Medicine at Mount Sinai, Baylor College of Medicine, University of California San Francisco/San Francisco Veterans Affairs Medical Center). All U19 participants were untreated and had to fulfill criteria for post-traumatic stress disorder (PTSD) for at least 3 months^23^.

The BerlinLCS cohort consisted of 173 children, who were recruited via child care centers, child and youth social services, child psychiatric departments, or pediatricians. Children were followed for 2.5 years with extensive psychometric and biological assessments. In addition, DNA from saliva samples was collected at five time points over the course of the study (every 6 months). Cases were victims of one or more of the following: physical abuse, physical neglect, and/or emotional maltreatment (MT) requiring intervention by social services.

The Helsinki Birth Cohort Study (HBCS)^24–26^ consisted of 77 men who were evacuated to Sweden or Denmark unaccompanied by their parents during the Second World War according to the Finnish National Archives’ register^27^. The controls were 74 men who were not evacuated, and who were matched to cases for birth year and father’s occupational status. These men donated blood for DNA samples in a clinical study in 2001–2004. Information on these five cohorts is summarized in Table 1.

**Table 1:** Demographic overview of cohorts.

Cohort	*N*^a^	Mean age (SD)	Sex (male)	Ethnicity	Assessment of CA	CA *N*^a^ (%)	Tissue	Methylation array
GRADY	309	42.08 (12.92)	25.56%	African American	Self-report	148 (47.90%)	Whole blood	450K
PReDICT	363	39.83 (11.50)	39.67%	Mixed	Self-report	164 (45.18%)	Whole blood	450K
U19	78	39.27 (12.10)	0.00%	Mixed	Self-report	66 (84.62%)	Whole blood	450K
BerlinLCS	173	4.23 (0.79)	52.60%	Caucasian	Documented	86 (49.71%)	Saliva	EPIC
HBCS	151	63.5 (2.8)	100%	Finnish	Documented	77 (51.00%)	Whole blood	450K

In GRADY, PReDICT, and U19 CA refers to moderate-to-severe ranges of CTQ scores for either sexual, physical, or emotional abuse (GRADY *n* = 77, PReDICT *n* = 79, U19 *n* = 14) or to moderate-to-severe scores in at least two abuse groups (GRADY *n* = 71, PReDICT *n* = 85, U19 *n* = 52), in BerlinLCS CA refers to maltreatment and in HBCS to evacuation and separation from parents in World War II.

*SD* standard deviation, *CA* childhood adversity.

^a^*N* = sample size.

### DNAm data

DNAm was measured by Illumina Infinium HumanMethylation450K BeadChips in GRADY, PReDICT, U19, and HBCS and by the Infinium MethylationEPIC BeadChip for BerlinLCS (for this study we focused on the baseline methylation levels). Beta values were normalized using functional normalization^28,29^. Batch effects were removed using ComBat^30^ with the sva package^31^. Subsequently, all CpGs on sex chromosomes and CpGs with SNPs in the probe sequence were removed. In addition, probes were removed if the detection *p* value was >0.01 in at least 25% of the samples, the probe contained SNPs in the single base pair extension or CpG position, the probe had missing beta values, or was a cross-reactive probe^32^. The Houseman method was used to estimate blood cell type composition^33^. Saliva cell counts for the BerlinLCS cohort were computed according to Smith et al.^34^. Smoking scores in each cohort were calculated as described by Elliott et al.^35,36^. For the BerlinLCS cohort, we computed a prenatal smoking exposure according to Richmond et al.^37^.

### Genotype data

#### DNA isolation and SNP genotyping

In all cohorts, except BerlinLCS, DNA was isolated from blood samples (GRADY: using either the ArchivePure DNA Blood Kit (5 Prime, Gaithersburg, MD, USA) or E.Z.N.A. Mag-Bind Blood DNA Kit (Omega Bio-tek, Norcross, GA, USA), U19: using the PerkinElmer Chemagic 360 extraction robot). In BerlinLCS, DNA was isolated from saliva samples. Genome-wide SNP genotyping was performed using Illumina OmniQuad (GRADY), HumanOmniExpress BeadChips (PReDICT and U19), Illumina GSA-24 v2.0 BeadChips (BerlinLCS), and Illumina 610k chips (HBCS, modified Illumina 610k chip by the Wellcome Trust Sanger Institute, Cambridge, UK).

#### Quality control and imputation

Quality control was performed in PLINK^38^ independently in all cohorts. Samples with low genotyping rate (<98%) were removed. SNPs with high rate of missing data (>2%), significant deviation from the Hardy–Weinberg equilibrium (HWE, *p* < 10^−5^), or a low minor allele frequency (MAF < 5%) were excluded from further analyses. Afterward, additional SNPs were imputed using IMPUTE v2^39^, the 1000 Genomes phase III sample served as reference panel^40^. Imputed SNPs with a low information content metric (<0.8), significant deviation from the HWE (*p* < 10^−5^), or low MAF (<5%) were excluded. In the HBCS cohort, genomic coverage was extended by imputation using the 1000 Genomes Phase I integrated variant set (v3/April 2012; NCBI build 37/hg19) as the reference sample and IMPUTE v2. Before imputing, the following quality control filters were applied: SNP clustering probability for each genotype > 95%, call rate > 95% for individuals and markers (99% for markers with MAF < 5%), MAF > 1%, and HWE *p* > 10^−06^. Moreover, heterozygosity, sex check, and relatedness checks were performed and any discrepancies were removed. Imputed genotype probabilities were converted into best-guessed genotypes using a threshold of 0.90. SNPs were pruned to a reduced subset of approximately independent SNPs using a repeated sliding window (window size of 100 kb, 5 kb shift at the end of each step) procedure with a pairwise SNP *R*^2^ threshold of 0.2^38^.

### Environmental data

#### Self-reported childhood trauma: childhood trauma questionnaire (CTQ)

The CTQ is a psychometrically validated assessment of physical, sexual, and emotional child abuse and neglect, using 28 self-report items^41^. Participants in GRADY, PReDICT, and U19 were classified into three groups based on established cutoff scores for moderate-to-severe exposure levels for each type of childhood abuse (CA; ≥10 for physical abuse, ≥8 for sexual abuse, ≥13 for emotional abuse)^41^. The first group of participants scored in the none-to-mild range for sexual, emotional, and physical abuse and was classified as negative for exposure, the second group scored in the moderate-to-severe range for either sexual, physical, or emotional abuse, and the third group scored moderate-to-severe in at least two abuse groups.

#### Verified childhood trauma

For BerlinLCS, maltreated (physical abuse, physical neglect, or emotional MT) children were recruited via child welfare offices, child and youth social services, child psychiatric departments, or pediatricians and corroboration/details of MT exposure was obtained by caretaker report. Assessment and coding of maltreatment was based on^42^ Of the 173 children, 86 were victims of MT. The 87 children in the control group presented with no MT, and no other significant stressors as assessed with the preschool age psychiatric assessment^43^.

#### Childhood adversities in the HBCS

In the HBCS sample that was used for this study, 77 individuals had been evacuated to Sweden or Denmark unaccompanied by their parents during the Second World War according to the Finnish National Archives’ register. This experience was used as CA in comparison to the other 74 individuals of the sample, who had not been evacuated.

### Statistical analyses

All statistical analyses were performed in each cohort independently using R version 3.5.2.

#### Identification and characterization of overlapping variable methylated CpGs

In order to correct DNAm levels for known confounders, these were regressed out of the beta values using linear regression separately for each cohort. Covariates were defined as sex, age, blood cell counts^33^, or saliva cell counts for BerlinLCS^34^, smoking scores, and genotype principal components to account for population stratification (GRADY: the first two PCs, PReDICT, and U19 and BerlinLCS: the first five PCs, HBCS: the first three PCs). Using residuals from these models, the median absolute deviation (MAD) was estimated per CpG as a robust measure of DNAm variability within each cohort. The MAD score is preferred for this purpose as it is not driven by outliers. We tested the 80th, 85th, 90th, and 95th percentile as cutoffs of the MAD score. The 80th percentile cutoff resulted in 45,962 variably methylated probes (VMPs) overlapping among GRADY, PReDICT, and U19. These VMPs were selected for initial analysis and defined as overlapping VMPs. For a sensitivity analysis, we further regressed out cohort-specific covariates (anxiety score in U19; depression score in GRADY, PReDICT, and U19; PTSD score in GRADY and U19). Addition of these covariates did not influence the results.

#### Explaining variability of VMPs

To assess to what extent genotype, environment (CA), genotype and environment, as well as genotype–environment interaction contributed to variation in VMPs, four different linear regression models (1–4) were tested for each overlapping VMP in each cohort to identify the model with the largest adjusted *R*^2^.

1. Environment model (CA): VMP ~ covariates + CA.
2. Genotype model (G): VMP ~ covariates + G_*i*_.
3. Additive model (G + CA): VMP ~ covariates + G_*i*_ + CA.
4. Interaction model (G × CA): VMP ~ covariates + G_*i*_ + CA + G_*i*_ × CA.

VMP represents the uncorrected beta value of the identified variable CpG site described above. For models (2–4), G_*i*_ is a SNP-genotype coded by the minor allele count (0, 1, 2); all pruned SNPs in a *cis* window of ±1 MB around the VMP were tested sequentially and the SNP that presented with the largest adjusted *R*^2^ was selected for further analysis. Covariates are the DNAm confounders and cohort-specific covariates described in the preceding section. In models (3) and (4) all possible SNP and CA combinations were tested

The model with the largest adjusted *R*^2^ value, explaining the most variance across (1)–(4), was chosen as the best model for that VMP.

A work flow of the general procedure is depicted in Supplementary Fig. 1.

**Fig. 1:**
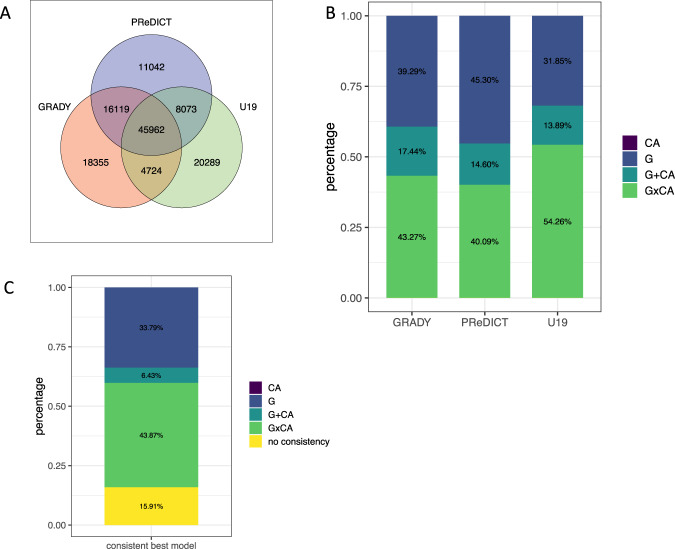
Overlapping VMPs between GRADY, PReDICT, and U19. Venn diagram of overlapping VMPs for GRADY, PReDICT, and U19 at a MAD score threshold at the 80th percentile (**a**). Distribution of the best models explaining variation in DNAm across the three adult cohorts. Percentage of overlapping VMPs (*n* = 45,962) best explained by G, CA, G + CA, or G × CA in each cohort using the highest adjusted *R*^2^ (**b**). Consistent best models across three adult cohorts. Percentage of overlapping VMPs (*n* = 45,962) best explained by the same model type (G, CA, G + CA, or G × CA) in at least two cohorts (**c**).

#### Mapping VMPs to genomic regions

VMPs were mapped to their genomic location using the R-packages *minfi*^28^ and *ChIPseeker*^44^ and to their corresponding ChromHMM states based on histone ChiP-Seq peaks from the Roadmap Epigenomics project derived for blood cells (http://egg2.wustl.edu/roadmap/data/byFileType/peaks/consolidated/broadPeak/).

Enrichment tests were performed using Fisher’s tests. The significance levels were set using Bonferroni correction according to the number of performed tests.

#### Gene-set enrichment analysis

VMP sites were mapped to their closest genes using the matchGenes function in the R-package bumphunter^45^. Gene-set enrichments were tested using FUMA’s GENE2FUNC v1.3.5^46^ setting the FDR adjusted *p* values for enrichment to 0.05 and considering Gene Ontology (GO) terms as well as tissue-specific transcripts derived from GTEx v6^47^. A minimum number of ten genes had to overlap with the specific gene set. We compared enrichments for stable across age G × CA CpGs (variably methylated CpGs with the best model being G × CA across all cohorts, *n* = 1400) and stable in adults G × CA CpGs (variably methylated CpGs with the best model being G × CA in the three adult cohorts, but not in the other two cohorts, *n* = 670). We used the group of inconsistent CpGs (*n* = 5652) that showed different best models across all three adult cohorts as control. For the enrichment, we created ten random subsets of genes mapping to these inconsistent CpGs and equal in size to the number of genes mapping to stable across age G × CA CpGs (*n* = 1123 genes).

## Results

### VMPs in adults with self-reported retrospective CA

We first assessed which of the four models (CA, G, G + CA, and G × CA) explained most of the DNAm variability in the three adult cohorts (see Table 1) that used the CTQ for retrospective assessment of CA. CpGs with a MAD score larger than the 80th MAD percentile and overlapping between GRADY, PReDICT, and U19 were defined as overlapping VMPs (*n* = 45,962 VMPs, see Fig. 1a). As previously described^17^, VMPs were enriched for distinct genomic features, including intergenic regions (*p* < 2.20 × 10^−16^, OR = 1.65, Fisher’s test) and enhancers (*p* < 2. 20 × 10^−16^, OR = 1.87, Fisher’s test).

We examined whether interindividual differences in DNAm levels of overlapping VMPs were better explained by genotype in *cis* (defined as 1 MB window around the specific VMP), by CA (E), or by additive or interaction effects of *cis* genotype (G) and CA together. For each cohort, we compared the adjusted *R*^2^ of four regression models (CA, G, G + CA, G × CA) to find the model which best explained DNAm variation in VMPs. The adjusted *R*^2^ is well suited to determine the most predictive model as it adjusts for the number of parameters in the model and only increases if the inclusion of these parameters also increases the model fit^48^. In all cohorts, the majority of VMPs was best explained by additive or interactive effects of G and CA (see Fig. 1b and Supplementary Fig. 2 for details) with CA alone being the best model only in very few VMPs.

**Fig. 2:**
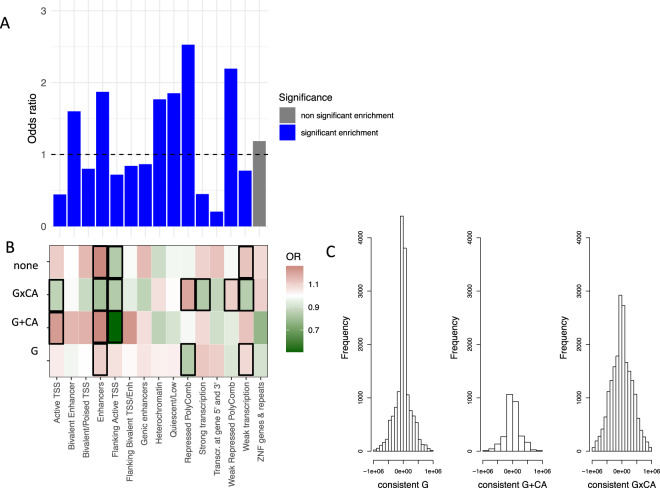
Enrichment of overlapping VMPs with regard to ChromHMM states. Histone mark enrichment for overlapping VMPs with regard to other 450K CpG sites (above panel) (**a**). Histone mark enrichment for overlapping VMPs (below) with at least two consistent best G, G + CA, or G × CA models against overlapping VMPs with no consistent models. Green color indicates depletion, and red color indicates enrichment. Thick black lines around the rectangles indicate significant enrichment/depletion based on Fisher’s tests and a Bonferroni threshold of *p* < 4.66 × 10^−04^. VMPs with at least two consistent best G × CA models were enriched in repressed Polycomb (*p* = 5.48 × 10^−19^, OR = 1.19, Fisher’s test) (**b**). Average distance between SNP and CpG site for consistent best G (left), consistent best G + CA (middle), and consistent G × CA models (right). CpGs with at least two consistent G × CA models presented with a significantly longer distance between SNP and VMP than VMPs with other consistent models (mean absolute distance = 271.83 kb, *p* < 2.20 × 10^−16^, Wilcoxon’s test) (**c**).

Over 80% of overlapping VMPs showed a consistent best model across at least two of the three cohorts (see Fig. 1c and Supplementary Fig. 3). As we based our results on pruned SNPs and as all cohorts had a different ethnic background, we matched the consistency based on best model for the same CpG only and did not require that the same SNPs be included in the model across cohorts. The majority of VMPs (43.87%) were consistently best explained by G × CA models. These results remained stable with inclusion of cohort-specific covariates, including symptoms severity (see Supplementary Figs. 4 and 5). For all cohorts, Δadj*R*^2^, i.e., the difference between the adjusted *R*^2^ of the best models to the adjusted *R*^2^ of the next best model, was highest for VMPs where G × CA was chosen as best model and significantly larger as compared to G and G + CA models (see Supplementary Fig. 6A–C, *p* < 2.2 × 10^−16^ for all cohorts, Wilcoxon’s test). VMPs with at least two consistent best G × CA models were enriched in repressed Polycomb (see Fig. 2a, b) and presented with a significantly longer distance between SNP and VMP than VMPs with other consistent models (see Fig. 2c).

**Fig. 3:**
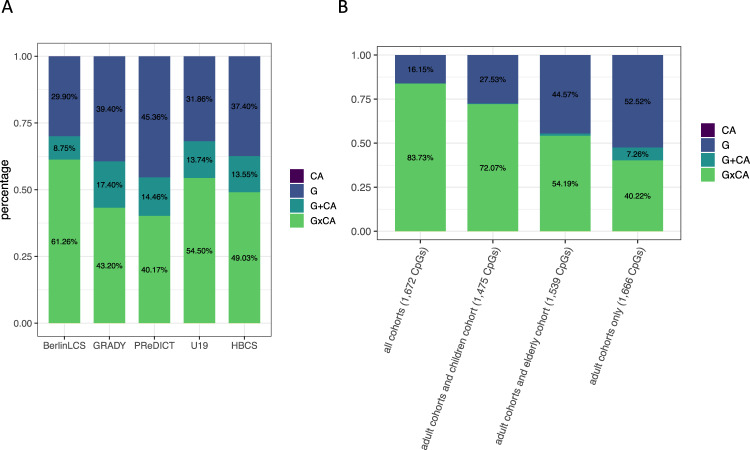
Distribution of the best models explaining variation in DNAm across the five cohorts. Percentage of overlapping VMPs (*n* = 36,091) best explained by G, CA, G + CA, or G × CA in each cohort using the highest adjusted *R*^2^ For HBCS, 49.03% of VMPs presented with best model G × CA, 37.40% with best model G, and 13.55% with best model G + CA, and for BerlinLCS, 61.26% of VMPs presented with best model G × CA, 29.51% with best model G, and 8.75% with G + CA (**a**). Consistency of best model explaining DNAm variability in VMPs across five cohorts. Percentage of VMPs with the same model explaining variation in DNAm best overlapping between the cohorts stratified by model type (**b**).

### VMPs across the life course and with documented adversity

To test if the identified combined effects of genotype and CA are stable across the life course and also observed with documented and not only self-reported adversity, we used two additional cohorts. The BerlinLCS cohort, consisting of 173 DNAm saliva samples of children aged between 3 and 5 years, of which 86 were recruited from social services and other child welfare centers due to MT or neglect. At the other end of the age spectrum is the HBCS, a cohort of 151 elderly individuals, of which 77 had been evacuated to Sweden or Denmark during World War II.

To base the comparison of best models across the developmental trajectory on the same CpG sites in all cohorts, we used the overlap of VMPs identified in GRADY/PReDICT/U19 and CpGs available in BerlinLCS as well as in HBCS. This resulted in 36,091 VMPs available in all five cohorts. Even with this more restricted set of VMPs, the best models remained combined models of G and CA (see Fig. 3a and Supplementary Fig. 7).

There were 1672 VMPs (5.4%) with a consistent best model across all five cohorts (see Fig. 3b). Among these stable VMPs, 83.73% had G × CA as the best model (*n* = 1400, “stable across age G × CA CpGs”).

In comparison, only 670 VMPs were consistently best explained by G × CA across the three adult cohorts but neither in the BerlinLCS nor in the HBCS (“stable in adults G × CA CpGs”). Both groups of CpGs were significantly enriched (*p* < 0.002 for both groups of CpGs) for eQTM sites^49^ as compared to all 450K CpGs (based on 10,000 randomly drawn CpG sets).

### Tissue specificity and functions of genes linked to stable G × CA CpGs

We annotated each VMP to the closest gene and used the list of unique genes to test for differences in gene-set enrichment using FUMA^45^. As a background set, we mapped the 36,091 VMPs which were used in the analysis across all five cohorts representing 10,308 unique genes.

**Fig. 4:**
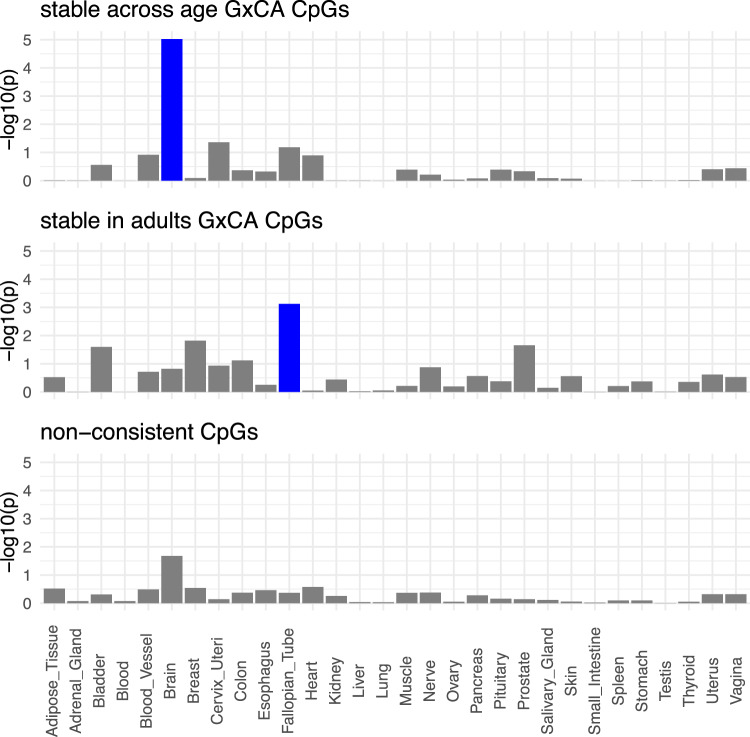
Enrichment of stable across age G × CA, stable in adults G × CA and non-consistent CpGs for GTEx upregulated gene sets. The *x*-axis denotes the −log10(*p* value), and the *y*-axis the specific tissue type. Significant enrichments (based on FDR correction of 0.05) are depicted in blue, and nonsignificant in gray. For the group of nonconsistent CpGs, we randomly picked ten subsets of genes mapping to nonconsistent CpGs, equal in number to the genes mapping to stable across age G × CA CpGs. None of these subsets presented with significant adjusted *p* values for enrichment. In the plot, median *p* values across all subsets are depicted.

We tested gene lists derived from stable across age G × CA CpGs and stable in adults G × CA CpGs for enrichment in differentially expressed gene sets across different tissues using the GTEx database^46^. The genes mapping to stable across age G × CA CpGs (1400 CpGs mapping to 1123 unique genes) were significantly enriched for genes specific to brain (FDR-corrected *p* value = 2.85 × 10^−04^) but not to other tissues. This enrichment for brain transcripts was not observed for genes mapped to stable in adults G × CA CpGs (670 CpGs mapping to 584 unique genes, FDR-corrected *p* value = 1.00 × 10^−01^). As control, we compared these to enrichments from random subsets from the list of inconsistent CpGs that showed different best models across all three adult groups (see Fig. 1c, *n* = 5652). We randomly picked groups of 1123 genes (which is the number of genes matching to stable across age G × CA CpGs) matching to these CpGs. None of these subsets showed significant tissue-specific enrichments (see Fig. 4).

Stable across age G × CA CpGs were significantly enriched for 24 GO terms, and stable in adults G × CA CpGs were significantly enriched for 35 GO terms in the biological processes categories (all FDR-corrected *p* values < 0.05). While some of these processes overlapped, stable across age G × CA CpGs were selectively significantly enriched in categories reflecting processes related to neuron development and synapse organization (see Supplementary Fig. 8). Stable across age G × CA CpGs were significantly enriched for the cellular component terms “neuron part” and “neuron projection” and the molecular function terms “DNA binding transcription factor activity,” “sequence specific DNA binding,” and “sequence specific double DNA binding” (all FDR-corrected *p* values < 0.05). Stable in adults G × CA CpGs had no cellular component or molecular function term significantly enriched. Non-consistent CpGs showed no significant consistent enrichments for any GO terms.

The analyses investigating tissue-specific gene expression as well as GO terms point to the fact that stable G × CA VMPs could have a distinct functional relevance, related to development and brain function.

## Discussion

In this study, we investigated the contributions of exposure to CA, genotype in *cis* as well as their additive and interactive effects on interindividual variability of DNAm in variable CpGs in peripheral tissues. Independent of the age of the cohort, we observed that models combining G and CA best explained DNAm variability in the majority of CpGs, suggesting that the extent of the combined impact on DNAm is similar for prenatal and postnatal adversity. For a set of 1400 VMPs, DNAm variability was best explained by G × CA across five independent cohorts, ranging in age from early childhood to late adulthood, suggesting a specific signature of CA independent of age. Interestingly, the genes mapping to these shared VMPs point to their potential relevance in development and brain function. Our results support the importance of including genotype when investigating environmental effects on DNAm, given that only G × CA but not CA alone unmasked a consistent pattern of DNAm variability across cohorts.

Our data are in line with previous EWAS results for CA that so far have yielded either inconsistent or negative results for the effect of CA alone^50,51^. Indeed, very few of the overlapping VMPs were best explained by environment independent of genotype (<1%). The majority of VMPs (~60–80%) were best explained by additive and interactive effects of genotype and environment together. To evaluate if we could also detect combined effects of CA and genotype in CpG sites which had previously been associated with CA, we used the publicly available results from Marzi et al.^50^ who studied the effect of early-life victimization on DNAm in peripheral blood in early adulthood and reported 63 CpG sites to be associated with victimization on an array-wide significant level. Testing these CpG sites in our adult cohorts revealed that CA alone was never the best model but that G × CA models were the most consistent best models for the majority (*n* = 20) of CpGs with consistent best models in at least two cohorts (*n* = 38).

The proportions of best G × CA models which we identified in our cohorts are analogous to the ~70% of variably methylated regions that were shown to be best explained by integrated genetic and prenatal environment effects in neonates^16,17^. Our findings corroborate that genotype acts as an important moderating influence on main environmental effects on DNAm also in the context of a postnatal stressor. In fact, G × CA was the model that best explained DNAm variance in the majority (83%) of CpGs that had the same best model across cohorts. The stability of the best model for these CpGs cuts across a large age range from early childhood to late adulthood, across tissue (blood and saliva), different DNAm variability thresholds, psychiatric diagnoses, as well as self-reported retrospective vs. verified CA. Additional studies in longitudinal cohorts with repeated measures of DNAm in the same individuals are needed to confirm such stability across time.

The VMPs with stable G × CA models mapped to genes with distinct functionality. In contrast to VMPs that only showed the G × CA model in all adult cohorts, but not more, the genes mapped to stable G × CA VMPs across five cohorts were enriched for transcripts specific to the brain (see Fig. 4) as well as to GO terms related to brain development and synapse function. Importantly, G × CA VMPs were also enriched for eQTMs, indicating that any factors influencing variability at these loci will have effects on gene transcription. Our samples size was underpowered to reliably detect consistent effect directions of SNP × CA interactions after correction for multiple testing. For this larger, ethnically homogenous cohorts will be necessary. Nonetheless, our results can highlight those CpGs that are most influenced by the combination of a genetic variant in *cis* and CA in a consistent manner across age, unmasking an epigenetic signature of CA.

Consistent with the previous literature^14^, *cis* meQTLs were clearly apparent in the five independent cohorts with diverse ethnic backgrounds. Although it is known that Caucasian and African American meQTLs significantly overlap, 14–45% shows specificity for ethnicity^52^. In our analysis, we found converging evidence that G × CA interactions best explain variability of DNAm across different ethnicities, but this does not exclude ancestry specific effects. To identify such specific interaction effects, larger samples for each ethnicity are required.

Finally, we want to note the limitations of this study. First, we restricted our analyses to specific DNAm array contents and to potentially functional CpGs, i.e., VMPs, so that we do not reflect every CpG tested on the array. Second, we used the adjusted *R*^2^ as main criterion for model fit as we were mainly interested in explaining variability of DNAm. A variety of other model selection criteria are available^53^ and which one to choose is an ongoing debate. Third, our analysis does not provide sufficient power to detect consistent effect directions after correction for multiple testing. In order to have sufficient power to assess specific SNP × CA effects surviving multiple testing correction larger, ethnically homogenous cohorts are necessary. All reported interactions are statistical interactions and limited to a *cis* window around the CpG site. Further experiments are required to assess whether these would also reflect biological/mechanistic interactions. Along the same lines, much larger cohorts will be needed to assess potential *trans* effects.

Furthermore, strategies to reduce the number of tests, i.e., SNPs, are needed. Possible methods include the prefiltering for functionally relevant SNPs using deep learning algorithm such as DeepSEA for instance^54^, or experimental approaches such as SNPs disrupting transcription factor binding or chromatin structure^55^. However, our results can highlight those CpGs that are most influenced by the combination of a genetic variant in *cis* and CA in a consistent manner across five cohorts and hence are environmentally sensitive. While our results highlight convergent effects of CA across ages, we did not have sufficient power to identify effects specific to certain forms of CA or neglect, or related to specific timing of the exposure. Our analysis provides a possible framework of how specific combined effects of genotype and environment on DNAm might be studied in the future.

In conclusion, in this study, we show that CA has a larger impact on DNAm in combination with genetic variation than by itself. Inclusion of information on genetic variation may thus help to uncover impact of environmental factors on epigenetic measures that would otherwise remain concealed. Such combined approaches could support to identify gene pathways relevant to risk or resilience following exposure to CA.

## Supplementary information

Supplemental Figure 1

Supplemental Figure 2

Supplemental Figure 3

Supplemental Figure 4

Supplemental Figure 5

Supplemental Figure 6

Supplemental Figure 7

Supplemental Figure 8

## Data Availability

Due to ethical issues and consent, datasets from PReDICT, U19, BerlinLCS, and HBCS analyzed during the current study are not publicly available. Interested researchers can obtain a deidentified dataset after approval from the respective study boards. Data requests may be subject to further review by the national register authority and the ethical committees (HBCS). The raw methylation data and all related phenotypes for the GRADY cohort have been deposited into NCBI GEO (GSE72680).
