# Supplementary figures and images for: Combined effects of genotype and childhood adversity shape variability of DNA methylation across age
Source: Transl Psychiatry. 2021 Feb 1;11:88. doi: 10.1038/s41398-020-01147-z (PMC7851167; doi:10.1038/s41398-020-01147-z)

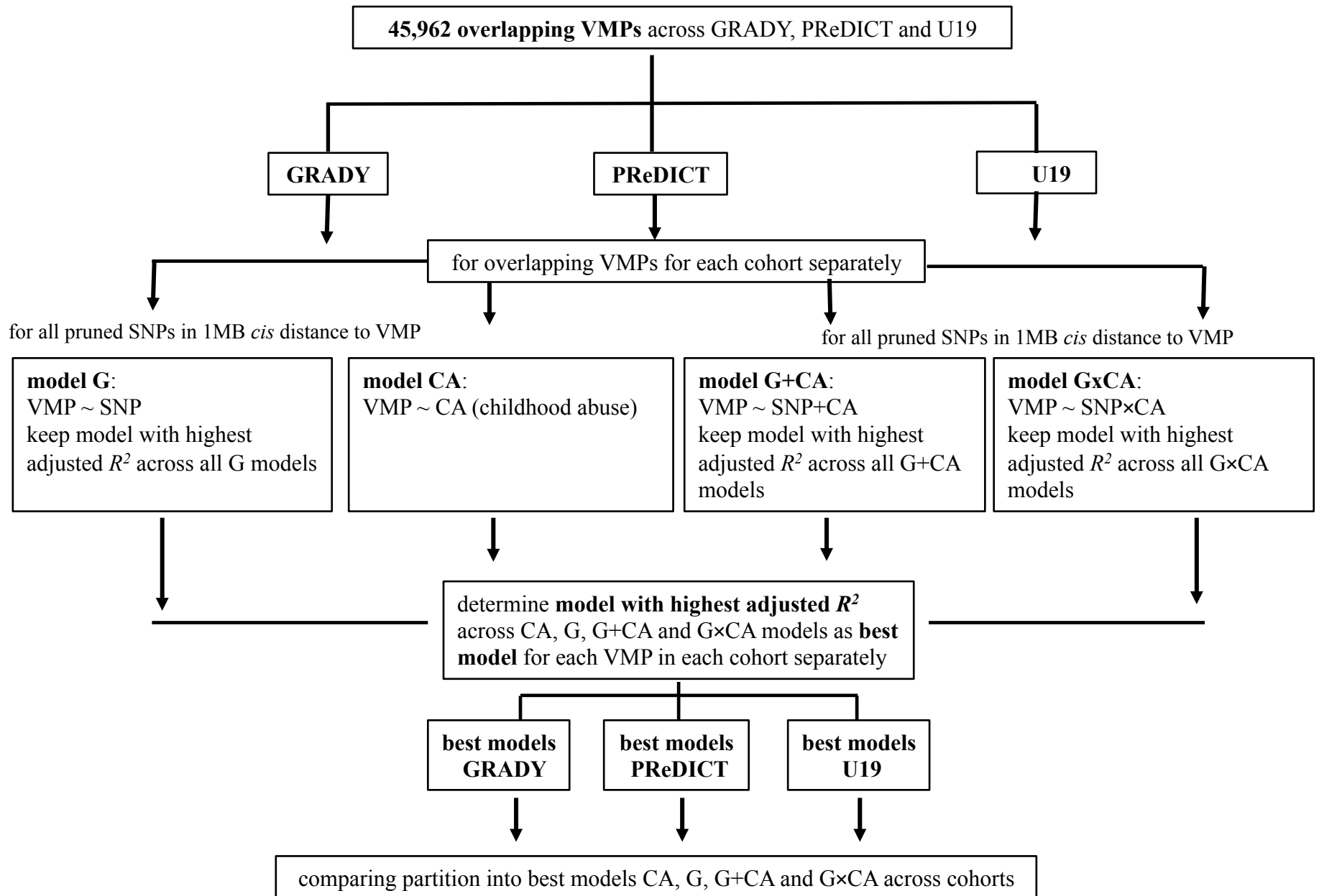

Suppl. Figure 1

## **Suppl. Figure 1: Work flow of the performed analyses**

Supplement: Supplementary file 2 — Supplemental Figure 2 [file 41398_2020_1147_MOESM2_ESM.pdf]
