# Supplementary material for: Combined effects of genotype and childhood adversity shape variability of DNA methylation across age
Source: Transl Psychiatry. 2021 Feb 1;11:88. doi: 10.1038/s41398-020-01147-z (PMC7851167; doi:10.1038/s41398-020-01147-z)
Supplement: Supplementary file 3 — Supplemental Figure 3 [file 41398_2020_1147_MOESM3_ESM.pdf]

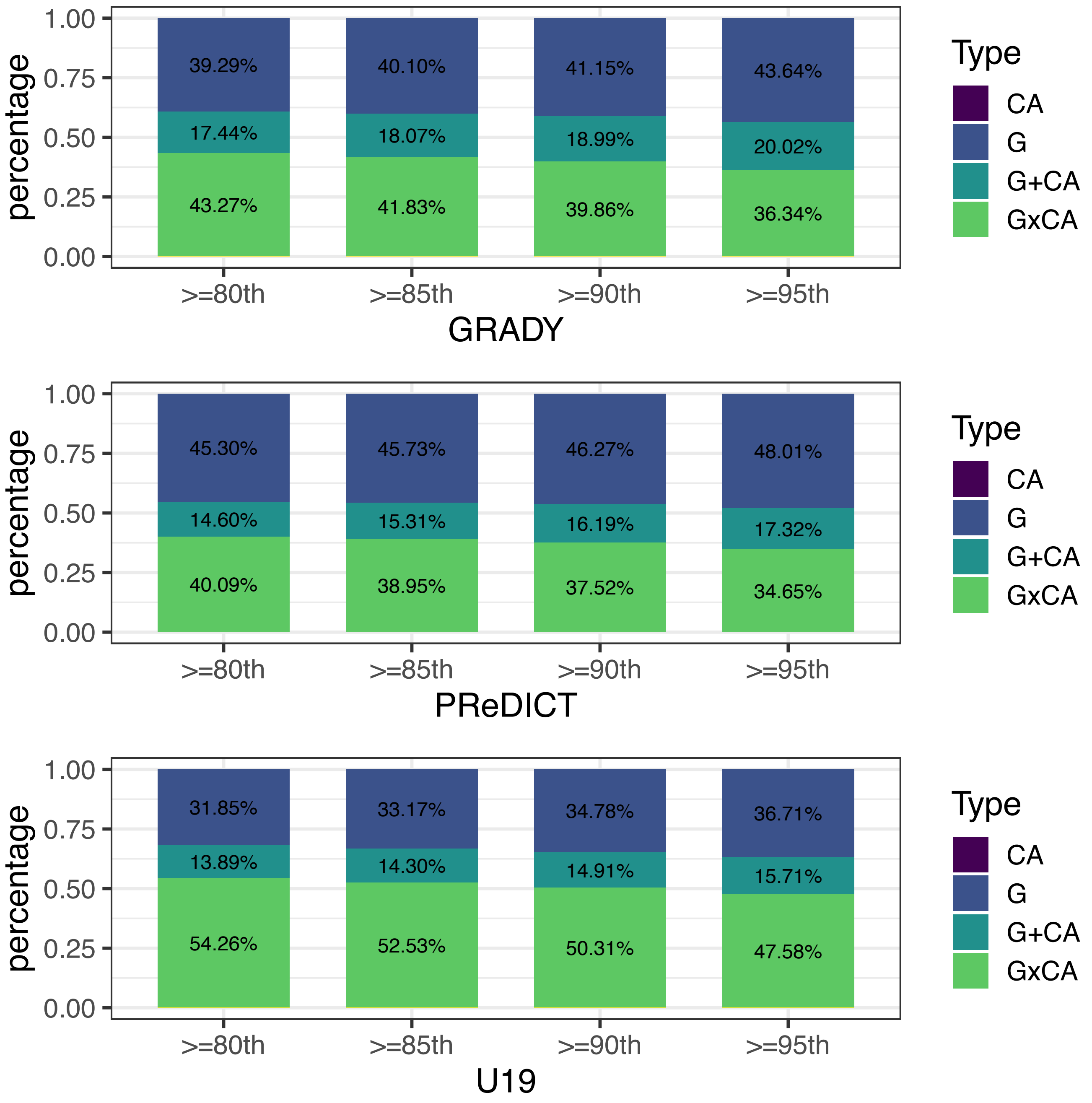

Suppl. Figure 2

Suppl. Figure 2: Distribution of the best models explaining variation in DNAm across the three adult cohorts. Percentage of overlapping VMPs (n=45,962) best explained by G, CA, G+CA or G×CA in each cohort using the highest adjusted R^2. Plots are stratified by MAD-score cutoff (>=80th percentile: n=45,962 sites; >=85th percentile: n=31,177 sites; >=90th percentile: n=20,360 sites; >=95th percentile: n=9,738 sites). The distribution of best models remained rather stable when varying the MAD-score threshold between the 80th and the 95th percentile with a small but significant increase of best model G with increasing methylation variability.
