# Supplementary material for: Combined effects of genotype and childhood adversity shape variability of DNA methylation across age
Source: Transl Psychiatry. 2021 Feb 1;11:88. doi: 10.1038/s41398-020-01147-z (PMC7851167; doi:10.1038/s41398-020-01147-z)
Supplement: Supplementary file 4 — Supplemental Figure 4 [file 41398_2020_1147_MOESM4_ESM.pdf]

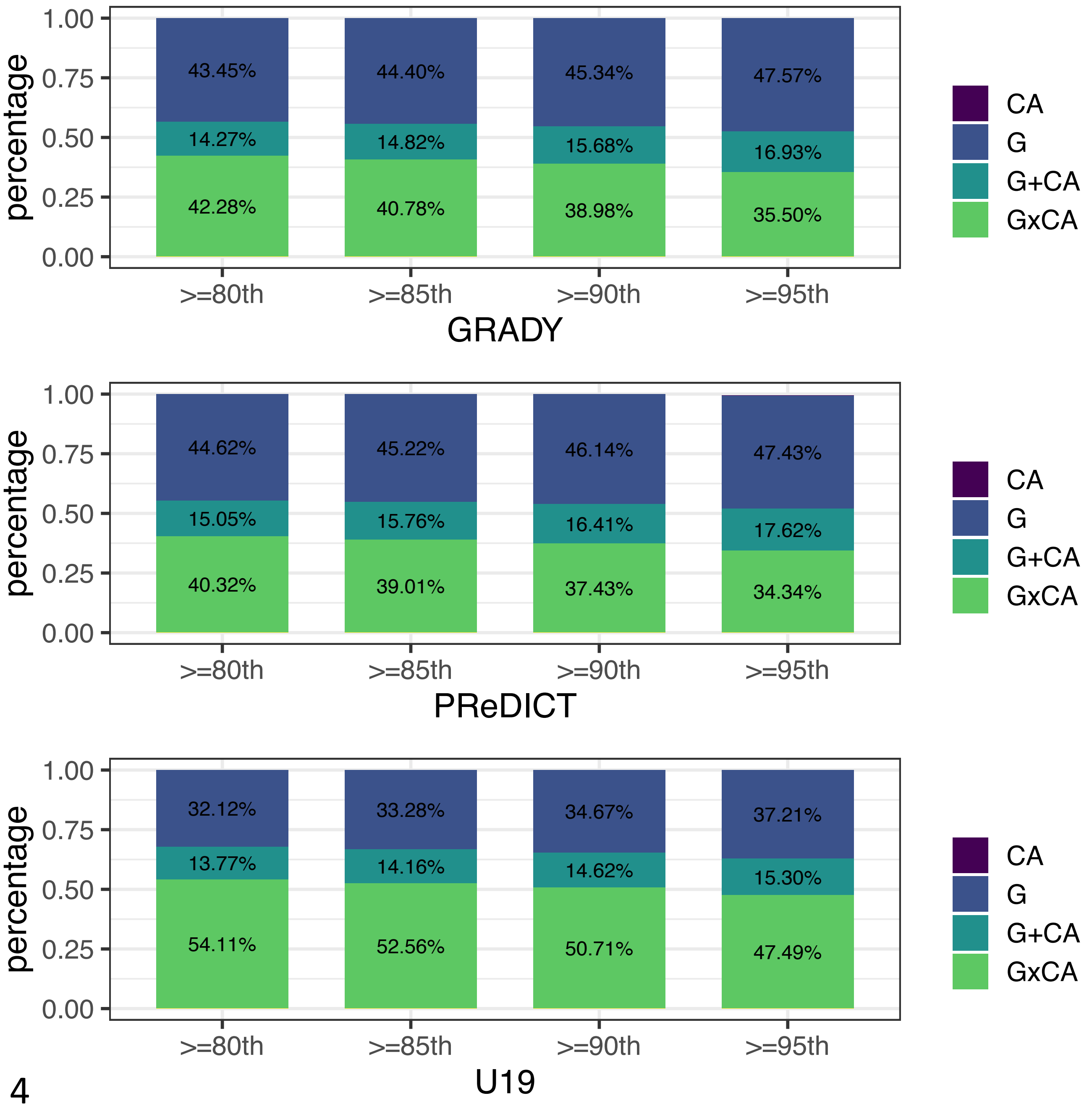


Suppl. Figure 4

**Suppl. Figure 4**: Distribution of the best models explaining variation in DNAm across the three adult cohorts, after correction for additional cohort-specific covariates. Percentage of overlapping VMPs (n=45,672) best explained by G, CA, G+CA or G×CA in each cohort using the highest adjusted $R^2$. Plots are stratified by MAD-score cutoff (>=80$^{th}$ percentile: n=45,672 sites; >=85$^{th}$ percentile: n=31,988 sites; >=90$^{th}$ percentile: n=20,196 sites; >=95$^{th}$ percentile: n=9,681 sites).
