# Supplementary material for: Combined effects of genotype and childhood adversity shape variability of DNA methylation across age
Source: Transl Psychiatry. 2021 Feb 1;11:88. doi: 10.1038/s41398-020-01147-z (PMC7851167; doi:10.1038/s41398-020-01147-z)
Supplement: Supplementary file 5 — Supplemental Figure 5 [file 41398_2020_1147_MOESM5_ESM.pdf]

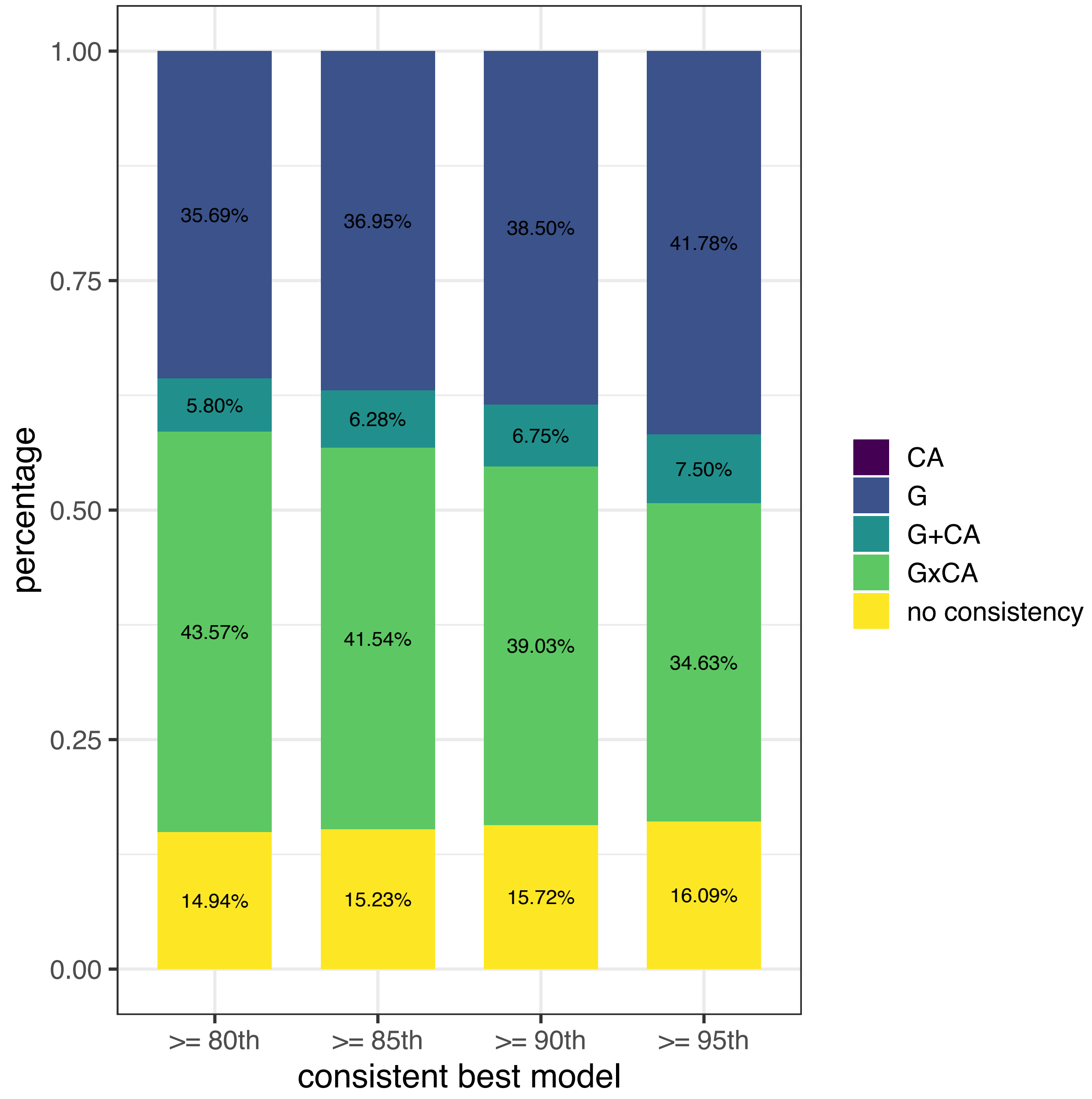

Suppl. Figure 5

Suppl. Figure 5: Consistent best models, after correction for additional cohort-specific covariates, across three adult cohorts stratified by MAD-score percentile cutoff (>=80th percentile: n=45,672 sites; >=85th percentile: n=31,988 sites; >=90th percentile: n=20,196 sites; >=95th percentile: n=9,681 sites).
