# Supplementary material for: Combined effects of genotype and childhood adversity shape variability of DNA methylation across age
Source: Transl Psychiatry. 2021 Feb 1;11:88. doi: 10.1038/s41398-020-01147-z (PMC7851167; doi:10.1038/s41398-020-01147-z)
Supplement: Supplementary file 6 — Supplemental Figure 6 [file 41398_2020_1147_MOESM6_ESM.pdf]

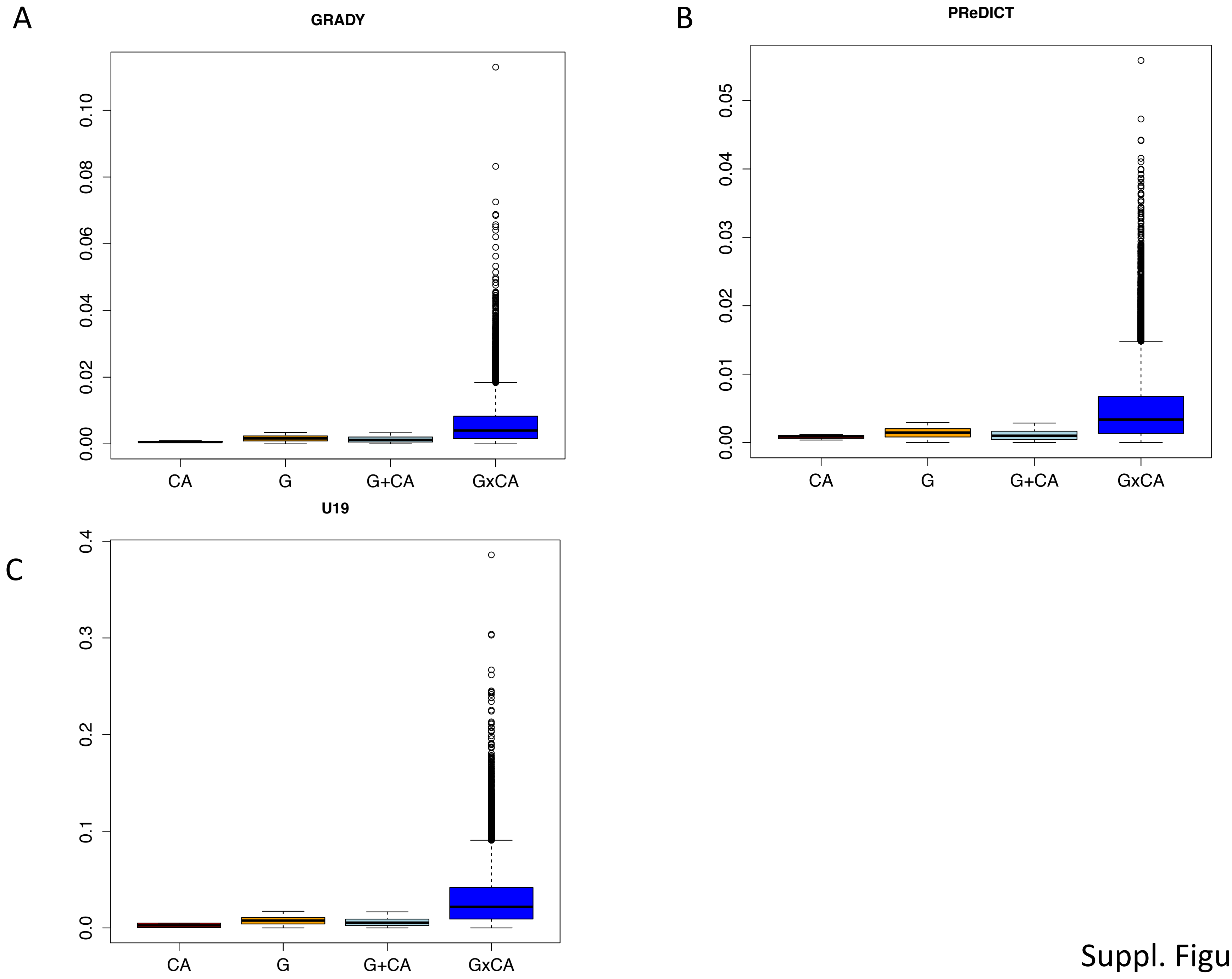

Suppl. Figure 6

Suppl. Figure 6: Boxplot of the differences in adjusted R^2 between the best model (highest adjusted R^2) and next best model (second highest adjusted R^2) in GRADY (**A)**, PReDICT (**B**) and U19 (**C**). The x-axis denotes the best model, the y-axis denotes the differences in adjusted R^2 between the best model (highest adjusted R^2) and next best model (second highest adjusted R^2).
