# Supplementary material for: Combined effects of genotype and childhood adversity shape variability of DNA methylation across age
Source: Transl Psychiatry. 2021 Feb 1;11:88. doi: 10.1038/s41398-020-01147-z (PMC7851167; doi:10.1038/s41398-020-01147-z)
Supplement: Supplementary file 7 — Supplemental Figure 7 [file 41398_2020_1147_MOESM7_ESM.pdf]

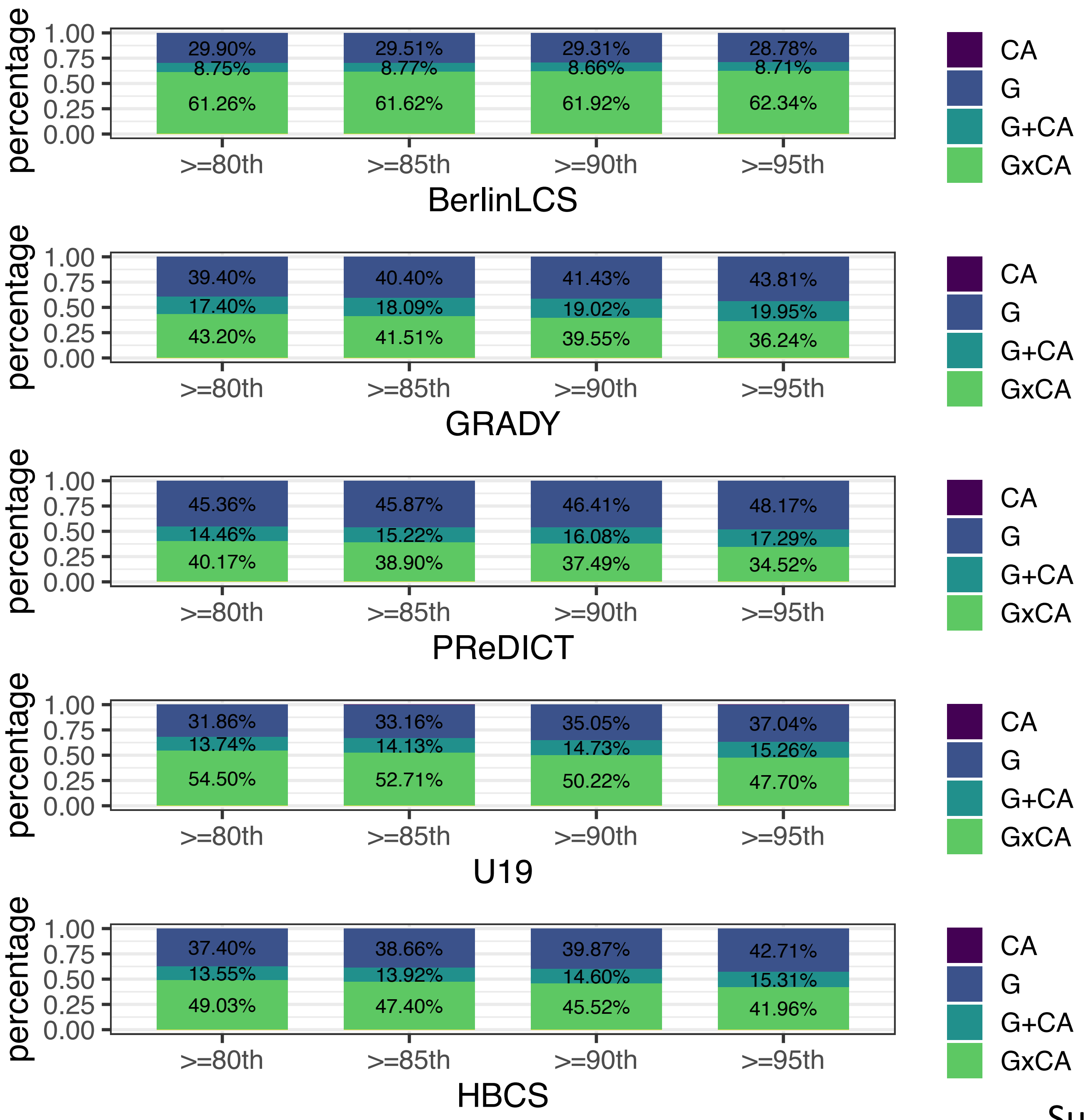

Suppl. Figure 7

Suppl. Figure 7: Distribution of the best models explaining variation in DNAm across the five cohorts. Percentage of tested VMPs (n=36,091 sites) best explained by G, CA, G+CA or G×CA in each cohort using the highest adjusted R^2 are depicted. Plots are stratified by MAD-score cutoff in the adult cohorts.
