# Supplementary material for: Combined effects of genotype and childhood adversity shape variability of DNA methylation across age
Source: Transl Psychiatry. 2021 Feb 1;11:88. doi: 10.1038/s41398-020-01147-z (PMC7851167; doi:10.1038/s41398-020-01147-z)
Supplement: Supplementary file 8 — Supplemental Figure 8 [file 41398_2020_1147_MOESM8_ESM.pdf]

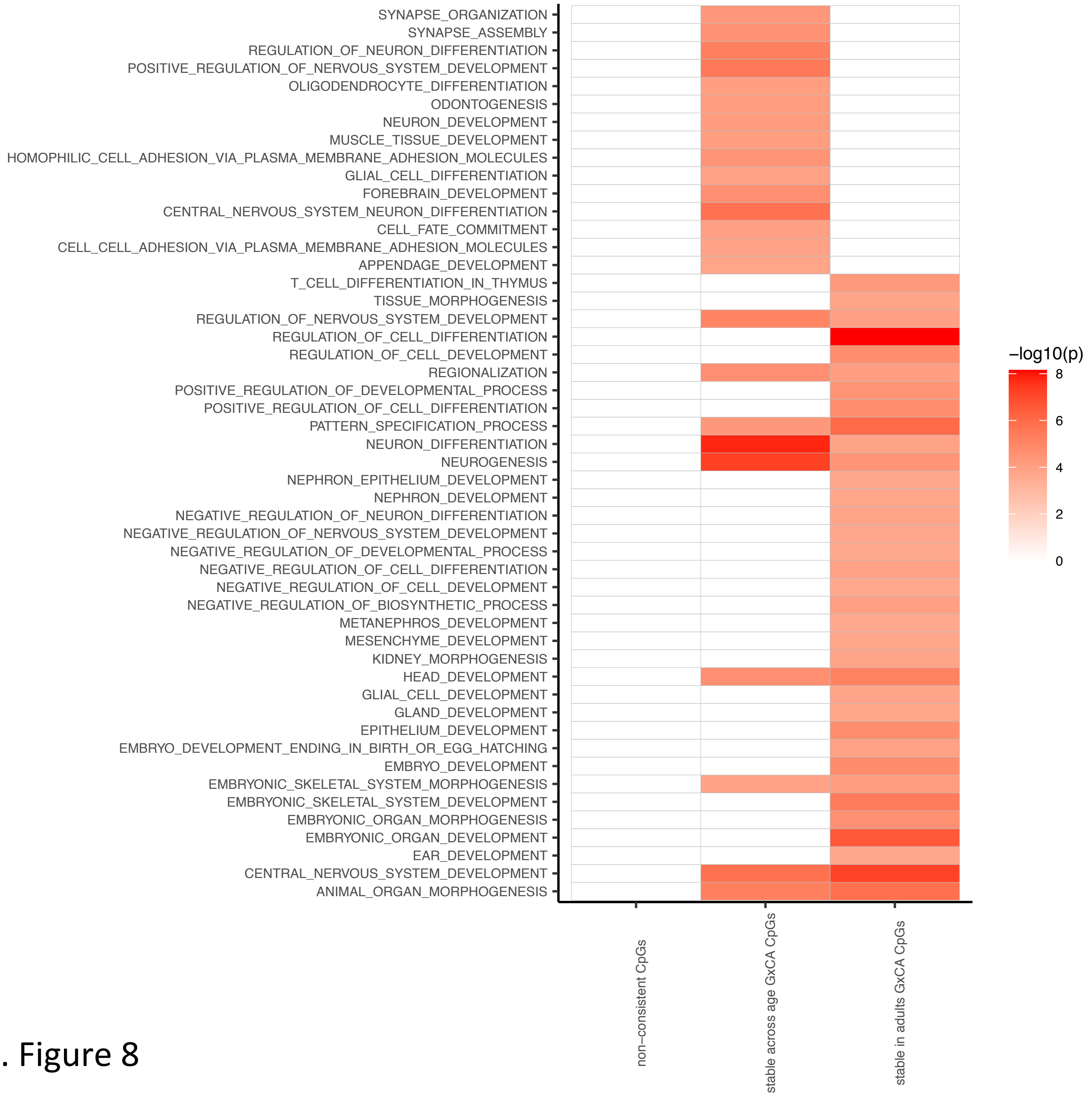

Suppl. Figure 8

Suppl. Figure 8: Enrichment of non-consistent, stable across age G×CA and stable in adults G×CA CpGs for GO biological processes. The x-axis denotes non-consistent, stable across age G×CA and stable in adults G×CA CpGs, the y-axis the GO biological process term. Only GO terms which are significantly enriched in at least one CpG group are depicted. The color indicates the –log10 (adjusted p-value). The non-conistent CpGs were not consistently enriched in any GO term (only 2 of the random subsets of non-consistent CpGs showed enrichment for GO terms, no term overlapped neither between the subsets nor with any of the significant GO terms for the stable G×CA CpGs).
